# Three-dimensional tracking of microbeads attached to the tip of single isolated tracheal cilia beating under external load

**DOI:** 10.1038/s41598-018-33846-5

**Published:** 2018-10-22

**Authors:** Takanobu A. Katoh, Koji Ikegami, Nariya Uchida, Toshihito Iwase, Daisuke Nakane, Tomoko Masaike, Mitsutoshi Setou, Takayuki Nishizaka

**Affiliations:** 1 Department of Physics, Faculty of Science, Gakushuin University, Toshima-ku, Tokyo 171-8588 Japan; 2 International Mass Imaging Center and Department of Cellular and Molecular Anatomy, Hamamatsu University School of Medicine, Hamamatsu, 431-3192 Japan; 3 Department of Anatomy and Developmental Biology, Graduate School of Biomedical and Health Sciences, Hiroshima University, Hiroshima, 734-8553 Japan; 4 Department of Physics, Tohoku University, Sendai, 980-8578 Japan; 5 Department of Applied Biological Science, Tokyo University of Science, Chiba, 278-8510 Japan

## Abstract

To study the properties of tracheal cilia beating under various conditions, we developed a method to monitor the movement of the ciliary tip. One end of a demembranated cilium was immobilized on the glass surface, while the other end was capped with a polystyrene bead and tracked in three dimensions. The cilium, when activated by ATP, stably repeated asymmetric beating as *in vivo*. The tip of a cilium in effective and recovery strokes moved in discrete trajectories that differed in height. The trajectory remained asymmetric in highly viscous solutions. Model calculation showed that cilia maintained a constant net flux during one beat cycle irrespective of the medium viscosity. When the bead attached to the end was trapped with optical tweezers, it came to display linear oscillation only in the longitudinal direction. Such a beating-mode transition may be an inherent nature of movement-restricted cilia.

## Introduction

Motile cilia of eukaryotic cells propagate bending waves and produce water flow over the cell surface. To produce unidirectional gross flow, cilia beat with a fast forward “effective stroke” and a slow backward “recovery stroke”. The trajectory of the ciliary tip in the effective stroke always takes a higher position (as viewed from the base) than that in the recovery stroke. This asymmetry is crucial to producing unidirectional flow, because the simple back-and-forth repetition of a rigid body cannot produce unidirectional medium flow^1^. Tracheal cilia, which function in viscous and highly variable conditions, maintain their periodic motion even when subjected to certain degrees of external perturbation^2–4^. To maintain the mucus-transportating function under various viscous conditions, cilia must undergo periodic and asymmetric shape changes during their effective and recovery beating phases in a wide range of viscous conditions.

In this study, we found that small polystyrene beads with sulfate residues predominantly attach to the tip of isolated cilia. Three-dimensional tracking of the bead allowed us to precisely track the position of the ciliary tip. The ciliary tip position during beating is a useful parameter to evaluate the asymmetry of the ciliary beating. We found that the cilium tip maintained a difference in height (termed “*z*-gap”) between the effective and recovery strokes under various conditions. Maintenance of a *z*-gap indicates a robust nature of ciliary asymmetry, which should be crucial to effectively producing flow under various conditions, as shown by our hydrodynamic model calculation.

To further examine the robustness that maintains a constant *z*-gap, we combined this system with optical trapping of the bead and analyzed the load-dependent behavior of cilia. This method enables precise position-control and observation of ciliary tip, as well as application of external force. Unexpectedly, while periodic oscillation persisted under bead-trapped conditions, the beating trajectory of the ciliary tip changed into a linear one moving only in the *z*-direction. This oscillation was composed of two different phases, which most likely corresponded to the effective and recovery strokes in beating cilia, although no significant curvature change was observed in the laser-trapped axonemes. This behavior indicates a robustness of cilia in beating in distinct effective and recovery phases even under extremely restrictive conditions.

## Results

### Assays to track single-cilium beating in 3-D

To analyze the beating properties of a single cilium, we isolated demembraned cilia from the murine trachea by the treatment with Triton X-100 detergent with gentle agitation. One end of the cilium non-specifically attached to the coverslip when the cilia suspension was introduced into a flow cell. The other end showed oscillatory movements when ATP was added. This movement, with a constant frequency at a given ATP concentration, resembled the beating of native cilia^5^. In separate observations with isolated and motility-reactivated axonemes, axonemes tended to attach to the glass surface by one end and bending waves almost always propagated from the attached end toward the other end (Supplementary Video-2). Thus it is most likely that the end attached to the glass surface corresponded to the ciliary base.

**Figure 1:**
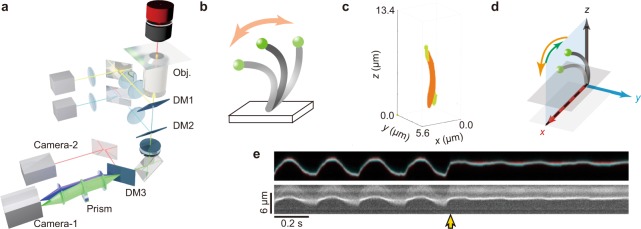
Schematics of the experimental setup. (**a**) Experimental setup. Yellow, pale blue and green lines represent optical paths of infrared laser, blue laser and the light emitted from a fluorescent bead, respectively. Blue and green cones in front of Camera-1 represent the paths of the light used to detect the *z*-displacement of the light emission from the bead. Orange line is the illumination by a deep-red LED used to visualize the mid-portion of a single cilium with differential interference contrast optics (DIC). The term Obj. represents the objective lens. DM1, DM2 and DM3 represent the dichroic mirrors for the infrared laser beam, for the blue laser beam and for the separation of Camera-1(for the DIC image) and Camera-2 (for the fluorescent image), respectively. (**b**) Schematic illustration of a sample. A single cilium isolated from the murine trachea (the grey curve) was immobilized onto the glass surface by one end, and a fluorescent bead (the green sphere) was attached to the other end. Not to scale. (**c**) A reconstructed 3-D display of sequential confocal fluorescence images of beads and a single cilium (each images are shown in Supplementary Fig. S1). Green and orange respectively indicate a 200 nm diameter bead and a cilium. A bead located near the ciliary base is a glass attached bead which indicates the surface of a coverslip. Note that bead image was displayed elongatedly along the *z*-axis, because the thickness of the point spread function of the confocal microscopy along the *z*-axis is larger than that of *x* and *y*-axes. (**d**) Definition of the three axes. The plane on which the bead trajectory moves most extensively was aligned along the *x*-axis, and the direction of the effective stroke (forward stroke moving faster) was taken as positive and the recovery stroke was taken as negative (moving slower). The *x-z* plane was set vertical to the plane of glass slide. (**e**) An example of a pair of kymographs, representing the spatial positions of a fluorescent bead image (*upper*) and the middle portion of the single cilium as a DIC image (*lower*). In a fluorescent bead image, two images separated by the prism were superimposed with different colors, red and blue. The yellow arrow indicates the onset of laser trapping of the bead (cf. Figs 4 and 5).

We found that sulfate-modified fluorescent beads (diameter, 0.2 μm) spontaneously attached to the free end of isolated cilia (Fig. 1b,c, and Supplementary Fig. S1 and Video-1). When cilia were suspended in the solution containing other types of beads, i.e., carboxylate-modified and amine-modified polystyrene beads, beads did not specifically attach to the end of the cilium. It indicates that the sulfate-modified fluorescent beads attached to the end of the demembranated cilium by hydrophobic interaction. Next, we tracked the position of a single bead using a 3-D tracking technique^6–8^. The light beam flux from the sample was split into two light paths (blue and green in Fig. 1a) by a wedge prism placed in front of a camera (Camera-1 in Fig. 1a). *Z*-directed movement was estimated from the relative displacement of the two images produced by the split beams, while *x*- and *y*-directed movements were determined from the average displacement of these images. This microscopy achieves nanometer accuracy, because it determines the localization of the bead (see Methods section in detail). Two laser beams of 488 and 1,064 nm were introduced into the microscope for fluorescent excitation and trapping of the bead, respectively.

**Figure 2:**
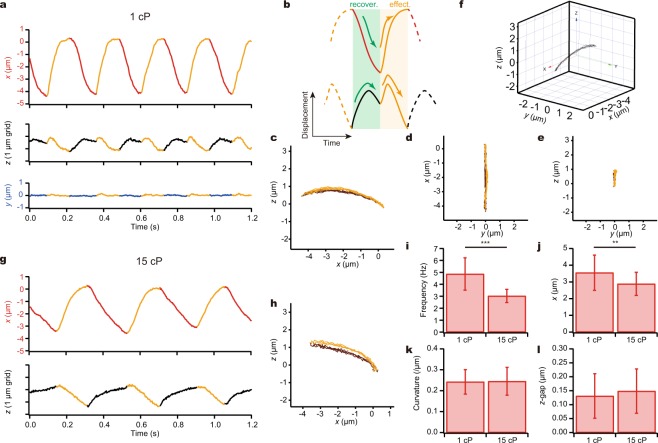
Movement of a bead attached to the tip of a demembranated and reactivated cilium. (**a**) A typical example of the time course of the bead movement in *x*, *y* and *z* directions at 100 µM ATP. (**b**) Schematic of a typical beating cycle in *x* (*top*) and *z* (*bottom*) directions. Oscillation is comprised of a recovery stroke and an effective stroke. (**a**,**c**–**e**,**g**,**h**) Orange lines represent the effective stroke phase. (**c**–**e**) Trajectories on the *xz*, *yx* and *yz* planes. In all the examples, the trajectory of the effective stroke was above that of the recovery stroke as seen in the *xz*-plot. (**f**) 3-D plots. (**g**) A typical example of the time course of the bead movement in the presence of methylcellulose. The medium viscosity was 15 cP. (**h**) Trajectories on the *xz*-plane at 15 cP. (**i**–**l**) Comparison of the bead movement at different viscosities. (**i**) Beating frequency. ***p < 0.001 two-tailed *t*-test. (*n* = 34 for 1 cP; *n* = 35 for 15 cP.) (**j**) The amplitude along the *x*-axis. **p < 0.01 two-tailed *t*-test. (**k**) Radius of curvature. (**l**) The *z*-gap between the effective and recovery strokes.

The coordination system used in this study is shown in Fig. 1d, where the *x*-axis is aligned parallel to the beating plane of the cilium. Ciliary bending in the positive direction corresponds to an ‘effective stroke’ (for definition, see Fig. 2a,b).

As clearly seen in the kymograph of a single bead attached to the free end of a cilium (Fig. 1e *upper panel*), the bead movement was roughly categorized into three phases: a fast effective stroke, a temporal drift with a lowered speed, and a recovery stroke after which the next effective stroke followed. The precise position of the bead was reconstructed in 3-D by a series of analyses (cf. Fig. 2), calibrated using a piezoelectric stage^6^ (see Methods section in detail). At the same time, the middle portion of a single cilium was checked in DIC images taken with another camera using a different wavelength of light (Camera-2 in Fig. 1), with the focusing plane shifted about 2.5 μm below the bead position. As a kymograph taken in the *x-y* plane (Fig. 1e, *lower panel*) shows, the trajectory of the middle portion was similar to that of the ciliary tip (Fig. 1e, *upper panel*) except for its smaller amplitude. The synchronous motions of the tip and the middle portions indicate that the two ciliary ends were stably attached to the bead and the glass surface and that no large-amplitude bending occurred in the middle portion of the cilium. Cilia apparently beat with a constant orientation keeping the concave part to the same side, and without such a large change in shape as might be expected if cilia underwent a large-scale undulating movement (Supplementary Fig. S2).

### 3-D trajectory of the ciliary tip in viscous environments

The experimental system described in Fig. 1 enables recording of the 3-D trajectory of a bead with nanometer-scale resolution^6,7,9–11^. After coordination alignment (cf. Fig. 1d), displacements along the *x*, *y* and *z* axes were determined as illustrated in Fig. 2a. In the *z*-direction, beating typically showed two peaks in each cycle. This is because the trajectories in both effective and recovery strokes were convex between the two end points (Fig. 2b). Multiple strokes stably traced the same trajectories as shown in an *xz*-trace (Fig. 2c). The repeatability between different strokes ensured that the attachment of the end of a cilium to the glass surface was strong enough and did not significantly fluctuate despite the movement of the cilium against surrounding fluid. The trajectory of effective strokes almost always took higher *z*-values than recovery strokes (cf. Fig. 2c).

To analyze the effect of medium viscosity, we increased the viscosity to 15 cP by adding methylcellulose. When viscosity was increased, beat frequency significantly decreased from 4.9 ± 1.4 Hz (mean ± s.d.) to 3.0 ± 0.5 Hz and the amplitude modestly decreased from 3.6 ± 1.4 μm to 2.9 ± 0.7 μm (*n* = 34 for 1 cP; *n* = 35 for 15 cP; Fig. 2i,j). Note that the beat frequency and amplitude at 1 cP were of the same order of magnitude as the beating of isolated cilia^5^. In contrast, the curvature of the trajectory in the *xz*-plane and the maximal *z*-gap between the trajectories of the effective and recovery strokes remained unchanged (Fig. 2k,l). The higher viscosity sensitivity of beat frequency than that of other waveform parameters was observed in previous studies on other types of cilia and flagella^12,13^.

### Estimation of the force and flow exerted by a single cilium

**Figure 3:**
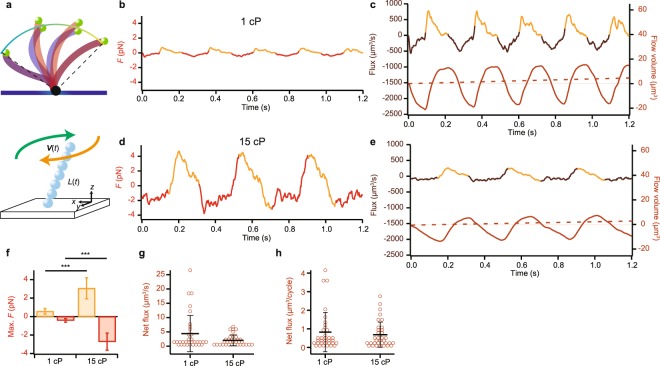
Estimation of the force and flux produced by a single cilium. (**a**) The adhesion point of the end of a cilium to the glass surface was approximated using the trajectory of the cilium (*top*). The cilium was represented by a linear chain of spherical beads (*bottom*). (**b**) Time course of the force acting on the surrounding fluid calculated from the observed bead trajectory (Fig. 2a). Beige and Red lines indicate the range where *F* > 0 and <0, i.e., indicating the force in the effective and recovery strokes. (**c**) Estimates of flux and flow produced by a single cilium in Fig. 2a. The total flow volume (*right axis*) is the time integral of the flux (*left axis*), and gradually increases with time. Dotted line indicates the net flux. (**d**) Time course of the force production in viscous medium calculated from the bead trajectory in the presence of methylcellulose (cf. Figure 2g). The medium viscosity is 15 cP. (**e**) Estimates of the magnitude of flux and flow produced by a single cilium at 15 cP (cf. Figure 2g). (**f**) Maximal forces produced during the effective and recovery phases. Beige and red bars indicate the effective and recovery strokes, respectively. ***p < 0.001 two-tailed *t*-test. (*n* = 34 for 1 cP; *n* = 35 for 15 cP) (**g**) The net (time-averaged) flux during one second. (**h**) The net (time-averaged) flux per one beat cycle.

To calculate the viscous drag force acting on a single cilium, as well as the net flow produced, we considered a simple model of ciliary movement. In this model, we assumed a single cilium as a linear chain of spherical beads, whose length varies between the effective and recovery beating phases (Fig. 3a). We assumed that the trajectories of the ciliary tip were contained in the surface of a sphere with its center at the ciliary attachment point on the glass surface (see Methods section for details; Fig. 3a). The length between this point and the ciliary tip defined the model length, which differs between the effective and recovery strokes, as indicated by distinct difference in the trajectory. Note that viscosity did not affect the estimated attached point, since the curvature of the trajectory did not change at two different viscosities (cf. Fig. 2k).

The maximal drag force in our model estimated based on ref.^14^ significantly increased at high viscosity from 0.57 ± 0.30 pN to 3.1 ± 1.1 pN in effective stroke and from 0.41 ± 0.20 pN to 2.7 ± 0.92 pN in recovery stroke (n = 34 for 1 cP; n = 35 for 15 cP; Fig. 3b,d,f). Net fluxes at low and high viscosities were calculated to be 4.4 ± 6.3 μm^3^/s and 2.0 ± 1.9 μm^3^/s, respectively (Fig. 3c,e,g). The net flux per one beat cycle was calculated to be 0.82 ± 1.0 μm^3^/cycle at low viscosity and 0.68 ± 0.67 μm^3^/cycle at high viscosity (Fig. 3h). We could not exclude the possibility that this model oversimplifies the ciliary shape. However, the flux calculated by this model is comparable to the flux per single cilium from the multi-cilia experiment (see Discussion and Methods sections).

### Trajectory of the ciliary tip under auxotonic conditions produced by optical trapping

**Figure 4:**
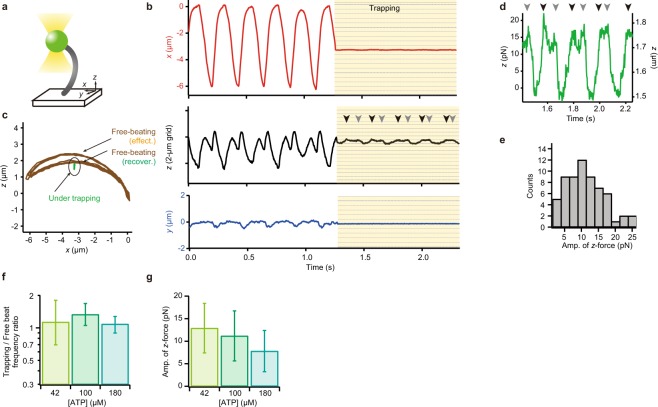
Optical trapping of the bead at the tip of the single cilium immobilized to the glass. (**a**) Schematic illustration of the experimental setup. Infrared laser light (yellow) was used for optical trapping. The bead used here (diameter, 1 µm) was larger than the beads used in other experiments. (**b**) An example of the time course of bead displacement in *x* (*top*), *z* (*middle*) and *y* (*bottom*) directions. The bead was trapped in a potential of 0.29 pN/nm in the *xy* plane and 0.10 pN/nm along the *z*-axis. (**c**) A typical example of trajectories in the *x-z* plane of an end-attached bead during beating. Brown, the trajectory under no load. Green, the trajectory of the bead under trapping. (**d**) Magnified view of time course of the *z*-axial trajectory under trapping conditions. Arrowheads in (**b**,**d**) indicate occurrence of two peaks during one cycle. (**e**) The maximal force estimated from the amplitude of oscillation of the bead (*n* = 62 trapping in 56 cilia). (**f**) Oscillating frequency under optical trapping. Columns in yellow-green, green, and emerald: 42 µM ATP, 100 µM ATP and 180 µM ATP (*n* = 11, 105 and 23). Bar chart shows geometric means ± geometric s.d. (**g**) The maximal force estimated from the amplitude of oscillation of the bead at different ATP concentrations.

To examine whether this asymmetric periodic motion of ciliary tip is maintained under a different type of perturbation, we trapped the fluorescent bead attached to the tip of cilium with an infrared laser (Fig. 4a). The present optical trapping confined a bead in a spring-like potential; in other words, it puts the cilium under an auxotonic condition, where the drag increases depending on the distance from the trapping center, but it is independent from the velocity of movement. Figure 4b shows a typical example of the trajectories of the end of a single cilium before and after optical trapping, obtained by tracking a 1-μm fluorescent bead attached to the tip. A larger bead was used here than in other experiments because a larger size of bead facilitates optical trapping. Trajectories of the bead were almost similar to those in Fig. 2a. When the spring constant of the potential was as weak as 0.29 pN/nm in the *xy* plane and 0.10 pN/nm along the *z*-axis, the beating amplitude along the *x*-axis significantly decreased (Fig. 1d, kymographs after the yellow arrow & Fig. 4b *top*). Interestingly, the tip kept oscillation, along the longitudinal axis of the cilium (Fig. 4b *middle* & green in 4c), displaying an almost linear trajectory. Importantly, however, it was not a simple back-and-forth motion. Two peaks were often observed during one cycle (Arrowheads in Fig. 4d) as in the movement of a tip in ciliary beating without trapping (cf. Fig. 2b *lower*), indicating that convex trajectories in both effective and recovery phases were maintained with a very small amplitude in the *x*-direction, even under the trapping condition.

Under the auxotonic loading conditions, as detailed in Materials and Methods, the maximal amplitude of oscillation in the *z*-direction was 154.7 ± 78.0 nm (*n* = 62). From this amplitude, we estimated that the maximal force produced by a single cilium was 11.1 ± 5.6 pN in the *z*-direction (Fig. 4e). This force did not change significantly at different ATP concentrations (Fig. 4g). Notably, the beat frequency slightly differed from that in free-beating cilia and this feature was constantly observed at various ATP concentrations (Fig. 4f).

**Figure 5:**
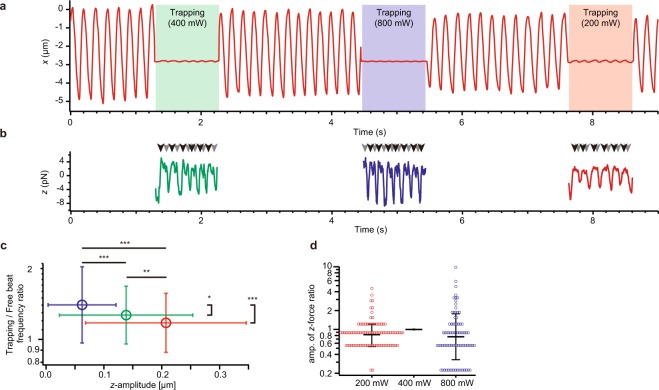
Optical trapping of the tip of the single cilium beating under various trapping stiffness. (**a**) An example of the *x*-displacement of a bead with or without trapping at different strengths. Green, blue and red areas respectively represent trapping with 0.074, 0.15 and 0.30 pN/nm potentials along the *z*-axis. (**b**) The force in the *z*-direction acting on the trapped bead. Arrowheads indicate the two peaks occurring during one cycle. (**c**) Change in frequency and amplitude with the change in optical trapping stiffness. Data with error bars in red, green and blue colors represent data sets taken under potentials with spring constants of 0.074, 0.15 and 0.30 pN/nm (*n* = 93, 95 and 79), respectively. *p < 0.05, **p < 0.01, ***p < 0.001 two-tailed *t*-test. Horizontal error bars and vertical error bars represent geometric means ± geometric s.d. and means ± s.d., respectively. (**d**) The ratios of the amplitude of force under 200 and 800 mW laser trapping to that under 400 mW laser trapping. Medial horizontal lines and error bars represent geometric means and geometric s.d., respectively.

To examine whether the maximal amplitude and the maximal force production in the *z*-direction vary with trapping stiffness, we trapped the same cilium at various laser intensities (Fig. 5a,b). Although the maximal amplitude of oscillation significantly varied with the trapping stiffness, the cilium produced almost the same maximal force. They also displayed similar frequency ratios between trapped and non-trapped conditions (Fig. 5c,d).

Finally, to examine whether the external force affects the cilium generated force, we bent or elongate step-by-step the same cilium along the *z*-axis during trapping by the optical tweezers combined with the piezo *z*-stage (Supplementary Fig. S3). Interestingly, the cilium exhibited the property that the amplitude of the force is decreased as the cilium is elongated. When the trapping center was displaced with +0.5 µm along the *z*-axis, the ratio of the amplitude of the force during elongate form and normal form was 0.63 (geometric mean). On the other hand, when the trapping center was displaced with −0.5 µm along the *z*-axis, the ratio of these forces was 1.13 (Supplementary Fig. S3c). A negative increment of the force was observed in 88% of cilia (*n* = 34, Supplementary Fig. S3d).

## Discussion

In this study, we developed a method to track the tip of a single beating cilium based on our finding that sulfate-modified fluorescent polystyrene bead spontaneously and predominantly attach to ciliary tips. Use of a 3-D tracking microscopy^6,7,9–11^ enabled high-speed tracking with a nanometer-scale precision. Furthermore, a combination with optical tweezers allowed us to measure the force on the bead while simultaneously monitoring its position. It remains to be clarified whether the sulfate-modified polystyrene bead could attach the tip of other types of cilia. Nevertheless, our method could be used for studying other kinds of cilia.

Using a simple model of ciliary beating and the parameters obtained from the ciliary tip trajectories, we estimated the force exerted by a cilium against the surrounding fluid (cf. Fig. 3b,d) and the magnitude of the resultant flow (Fig. 3c,e). The net flux caused by a single cilium we calculated (Q ~ 4 µm^3^/s) was comparable to the flux produced by a single cilium estimated using the data from previous studies on multiple cilia (~3 µm^3^/s; see Methods).

To examine the effect of load on ciliary movement, we changed the viscosity of the medium by addition of methylcellulose and found that the maximal *z*-gap was stable despite the absence or presence of methylcellulose (Fig. 2l). According to our model calculation, the net fluxes per one cycle did not differ either (Fig. 3h). These results suggest robustness in the production of asymmetric beating patterns necessary for effective flow generation, under various viscous conditions.

In previous studies with the sperm of *Chaetopterus*, *Ciona* and *Lytechinus*, the radius of curvature and/or the contour length of the bending portion decreased as the viscosity increased^13^. In our experiment, however, mouse tracheal cilia at high-viscosity retained almost the same curvature in the tip trajectory with a slight increase in the *z*-gap (Fig. 2k,l). This observation suggests that the high viscosity did not significantly change the waveform or affected the waveform in different manners between the effective and recovery strokes. Because the ratio of the ciliary length to the wavelength for the tracheal cilia is different from those of sperm flagella, there is a possibility that the effects of the viscous drag force are different between them. Also, the observed maintenance of asymmetric trajectory, which was found to be important for efficient flow production on the surface, seems to be a characteristic of mouse tracheal cilia. A previous study has suggested that the mouse tracheal cilium has a slight structural asymmetry around the nine outer doublets^15^. This asymmetry may be responsible for producing asymmetric beating patterns, as well as for the different response to viscosity in the effective and recovery strokes.

In our trapping experiments, the ciliary tip came to move only in the *z*-direction when trapped, while the oscillation persisted with an ~150 nm amplitude (cf. Fig. 4d). Since a ciliary axoneme is likely to maintain its length as long as its cytoskeleton is intact, the movement and force generation in the *z*-direction must be produced by the bending of the axoneme. The presumptive bending, estimated from the amplitude of the ciliary tip during trapping, is likely to have an amplitude of the order similar to that of the movement in the *z*-direction, i.e., about 0.2 µm. Detection of such small-amplitude bending in the middle portion of the cilium remains to be performed.

The maximal force along the *z*-axis showed a constant value (~11 pN) under a variety of conditions, except for the cases where the external longitudinal force was applied to the cilium (cf. Figs 4, 5 and Supplementary Fig. S3). On the other hand, the cilium-generated force along the *x*-direction was greatly decreased when the tip was trapped (cf. Fig. 4b). These unexpected results indicate that cilia can continue to beat when one end is immobilized and the other end is trapped by a spring-like potential that restricts beating. However, it is not understood why beating continues predominantly in the *z*-direction, even though optical tweezers restricted the bead movement in all directions. It may be that the ciliary axoneme, when the tip is trapped, has an inherent tendency to change its beating pattern from a normal beating with an asymmetric wave form to a pattern that could be detected only as a longitudinal oscillation at the tip. As a possible interpretation, considering the geometric clutch hypothesis^16–18^, we propose a model in which a cilium requires the change in the Global *t-force* in order to switch dynein bridges when the shape of the cilium is restricted almost completely. Thus, the cilium moves along the longitudinal direction during trapping in order to accumulate the longitudinal stress, because the longitudinal tension or compression are exerted on the dynein in the form of the Global *t-force*^16,17^. Indeed, the active force generated by the cilium changes when the external longitudinal force is applied (Supplementary Fig. S3). Whether the oscillation only in the *z*-direction is actually caused by such a hypothetical transition in ciliary beating mode, and if so, what is the mechanism underlying such beating-mode transition, are important future problems.

## Methods

### Mice

C57BL/6J mice were purchased from Sankyo Labo Service Corporation, Inc. (Tokyo, Japan). All mouse use experiments followed protocols approved by the Animal Care and Use Committees of Gakushuin University, license number 12. All methods were performed in accordance with the relevant guidelines and regulations.

### Isolation and preparation of cilia

Tracheae were taken from adult 8-10 week-old male mice, typically two mice in one preparation, and placed in the cold PBS solution kept in an ice bath. Debris of extra fat and connective tissues were immediately removed from tracheae by tweezers, and the trachea was subsequently divided into eight pieces with sizes of ∼2 mm to expose the ciliated epithelium. The isolation of cilia was performed based on the method reported previously^5,19^. These tissues were vortexed intermittently for 2 min in 500 µL volume of 20 mM Tris-HCl (pH 7.5), 50 mM NaCl, 10 mM CaCl_2_, 1 mM EDTA, 1 mM dithiothreitol and 0.1% Triton X-100. Cellular debris was pelleted by two rounds of centrifugation at 1,500 × *g* for 2 min. Cilia in the supernatant were subsequently collected by two rounds of centrifugation at 12,000 × *g* for 5 min and resuspended in 20 mM Tris-HCl (pH 8.0), 50 mM KCI, 4 mM MgSO_4_, 1 mM dithiothreitol, and 0.5 mM EDTA containing 1 tablet of protease inhibitor (cOmplete Mini EDTA-free; Roche) per 10 mL.

### Reactivation of single isolated cilia

A 1.3 μL volume of cilia suspension was incubated on a coverslip (3222 No.1; Matsunami Glass Industry) for 10-30 s, and a 0.7 µL volume of 0.03% sulfate-modified polystyrene beads (ϕ = 1 µm, yellow fluorescent, F8852; Invitrogen or ϕ = 0.2 µm, yellow fluorescent, F8848; Invitrogen) was subsequently infused. To reactivate the surface-immobilized cilia, a 2 µL volume of 200 µM ATP, 20 mM Tris-HCl, pH 8.0, 200 mM potassium acetate, 6 mM MgSO_4_, 1 mM dithiothreitol and 0.5 mM EDTA, was added and mixed. The viscosity of the solution was increased by dissolving 0–2% (w/v) methylcellulose (Methyl cellulose 15, 15 cP at 2% solution; Wako). Most of the cilia are laid on the coverslip. We sought for the cilium that sticks straight up from the coverslip that meets our experimental conditions. All the experiments were performed at 23.0 ± 0.1 °C.

### Microscopy

Single fluorescent beads attached to ends of cilia were visualized under an inverted microscope (Ti-E; Nikon Instruments) equipped with a 60× objective lens (CFI Plan Apo 60× TIRF 1.45 N.A.; Nikon Instruments), a single-mode fiber laser (λ = at 1,064 nm, YLM-1064-LP; IPG Photonics) with DM1 in Fig. 1a (ZT1064rdc-sp; Chroma Technology), a 488-nm laser (OBIS 488 LX; Coherent) with DM2 (zt488/561rpc; Chroma Technology), an emission filter (FF01-520/35; Semrock), an EMCCD camera (iXon^+^ DU860; Andor) as Camera-1, sCMOS (Zyla 4.2; Andor) as Camera-2, DM3 (Q650spxr; Chroma), a highly stable customized stage (Chukousha), and an optical table (RS-2000; Newport). The optical component for 3-D tracking has been described previously^6–8^. We acquired all the data with a time resolution of 2.82 ms (354.61 frames per second).

### Analyses

The positions of the two optically separated images of a bead from a single fluorescent bead were determined by 2-D Gaussian fitting as (*x*_1_, *y*_1_) and (*x*_2_, *y*_2_), and *x*, *y* and *z* were determined as (*x*_1_ + *x*_2_)/2, (*y*_1_ + *y*_2_)/2 and (*x*_1_ − *x*_2_)/2, respectively, with a resolution of nanometer. Relationship between the real displacement along *z* direction and (*x*_1_ − *x*_2_)/2 were determined with a calibrated piezo actuator (P-620.ZCL; Physik Instrumente GmbH & Co). Frequency in Figs 2i, 4f and 5c were estimated by the package function included in Igor Pro (WaveMetrics). We define the effective stroke as the stroke that higher velocity^20,21^.

### Model

Trajectories of the tip of a single cilium for ∼two seconds were approximated by the function exhibiting the spherical surface, and the coordinate origin was set to the center of the sphere assuming that the theoretical point of the other end of the cilium attached to the glass surface is the center of the sphere. To estimate this point, we fitted both effective and recovery trajectories by the function of the sphere. If the fitting results do not represent correct sphere orientation, we used the function of the sphere by fixing the center on the beating plane. The bead position was acquired and then smoothed using the fourth-order Savitzky-Golay method implemented in an analysis software, Igor Pro, to reduce spike-like noises in the force estimation (cf. Fig. 3b,d). The force *F* [pN] was calculated as *F* = −2π*ηL/*[ln*(L/*2*a)* + *γ*_⊥_] *V*, where *η* is the shear viscosity of the fluid, *a* = 0.1 μm is the radius of the cilium, and *γ*_⊥_ = 1.111 is the correction constant derived from Yamakawa’s model^14^. Other theories for viscous drag force on slender bodies give the same formula with different values of *γ*_⊥_, and are considered less accurate^22^. The flux *J* [μm^3^ s^−1^] was calculated as *J* = −2*zF*_*x*_/3πη, where *z* and *F*_*x*_ are the *z*-component of the tip position of the cilium and *x*-component of the total drag force, respectively. This equation is derived from the corresponding formula for a single bead in refs^23,24^, assuming that the drag force on each segment of the cilium linearly increases with its height from the surface. The flow volume was determined as the integral of the flux over a given window of time.

### Estimation of the flux

For rough estimation of the flux per single cilium from the multi-cilia experiment, we assumed that the flow speed is constant in *L*^3^ volume, where *L*^2^ is the area that cilia uniformly distribute. Total flux volume, *Q*, is *L*^2^*v*, where *v* is the one-directional flow speed measured in the experiment. As *L*^2^ is represented by *NA*, where *N* is the number of cilia per unit area and *A* is the area occupied by single cilium, *Q* is represented by *NAv*. Therefore, the flow volume driven by a single cilium is approximated by *q* = *Q*/*N* = *Av*. With the assumptions that the density of cilia in the tracheal ciliated cell is 6–8 per micrometer square^20^, the percentage of ciliated cells is ∼40% (ref.^25^) and the flow speed on the cell surface is ∼10 μm/s (ref.^15^), the volume of the medium displaced by a single cilium is roughly estimated to be (1/3 μm^2^) × 10 μm/s, i.e., ∼3 μm^3^/s.

### Trapping

In the experiment shown in Fig. 4, we set the trapping point lower than the trajectory of the effective stroke. This is a requirement for estimation of the active force produced by cilia, because the trapping point higher than the length of the cilium should straighten the cilium over its whole length, which may cause unnatural deformation of the cilium. We set the trapping point near the center of the beating trajectory in the *x*-*z* plane, at a point between 25% and 75% of the *x*-amplitude. Note that we confirmed that all cilia continued beating after they were released from trapping. In the experiment shown in Supplementary Fig. S3, during trapping, we moved the trapping center ± 0.5 µm along the *z*-axis using piezo *z*-stage (P-620.ZCL; Physik Instrumente GmbH & Co).

### TIRF and confocal microscope observation

Isolated cilia on the coverslip was stained by replacing Cy5-NHS ester (GE Healthcare) in infusion buffer [20 mM HEPES-NaOH (pH 8.0), 25 mM KCI, 100 mM potassium acetate, 5 mM MgSO_4_, 1 mM dithiothreitol and 0.5 mM EDTA]. After incubation for 5 min at room temperature, the adhesion point of the end of cilium was observed using TIRFM (the same microscope as the Microscopy in the Methods section) combined with a 561-nm laser (Sapphire; Coherent). Stained cilia were chemically fixed by replacing 4% (vol/vol) glutaraldehyde in infusion buffer. After incubation for 15 min, the 3-D image of the bead attached cilium was observed using a confocal microscope (FV1000; Olympus) equipped with a 100x objective lens (UPlanSApo, 1.4 N.A.).

## Electronic supplementary material

Supplementary Information

Supplementary Video 1

Supplementary Video 2
