# Supplementary material for: Three-dimensional tracking of microbeads attached to the tip of single isolated tracheal cilia beating under external load
Source: Sci Rep. 2018 Oct 22;8:15562. doi: 10.1038/s41598-018-33846-5 (PMC6197291; doi:10.1038/s41598-018-33846-5)
Supplement: Supplementary file 1 — Supplementary Information [file 41598_2018_33846_MOESM1_ESM.docx]

**Supplementary Information**

Three-dimensional tracking of microbeads attached to the tip of single isolated tracheal cilia beating under external load

Takanobu A. Katoh^1*^, Koji Ikegami^2,3^, Nariya Uchida^4*^, Toshihito Iwase^5^, Daisuke Nakane^1^, Tomoko Masaike^5^, Mitsutoshi Setou^2^, & Takayuki Nishizaka^1*^

^1^Department of Physics, Faculty of Science, Gakushuin University, Toshima-ku, Tokyo 171-8588, Japan. ^2^International Mass Imaging Center and Department of Cellular and Molecular Anatomy, Hamamatsu University School of Medicine, Hamamatsu 431-3192, Japan. ^3^Department of Anatomy and Developmental Biology, Graduate School of Biomedical and Health Sciences, Hiroshima University, Hiroshima 734-8553, Japan. ^4^Department of Physics, Tohoku University, Sendai 980-8578, Japan. ^5^Department of Applied Biological Science, Tokyo University of Science, Chiba 278-8510, Japan.

Correspondence should be addressed to T.N. (email: takayuki.nishizaka@gakushuin.ac.jp), N.U. (uchida@cmpt.phys.tohoku.ac.jp) and T.A.K (takanobu.a.katoh@gmail.com).


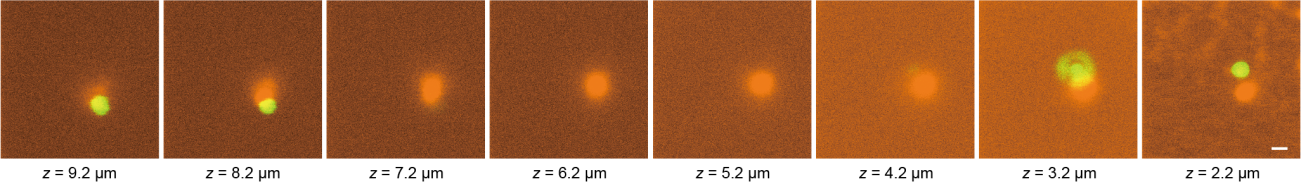


**Supplementary Figure 1** Sequential confocal fluorescence images of beads and a single cilium. Green and orange respectively indicate a 200 nm diameter bead and a cilium. Green spots in images taken at sections located at *z* = 9.2 µm and *z* = 8.2 µm represent the bead attached to the free end of the cilium. Green spots in images taken at sections located at *z* = 3.2 µm and *z* = 2.2 µm represent the bead attached the surface of the coverslip. Scale bar, 0.56 µm.


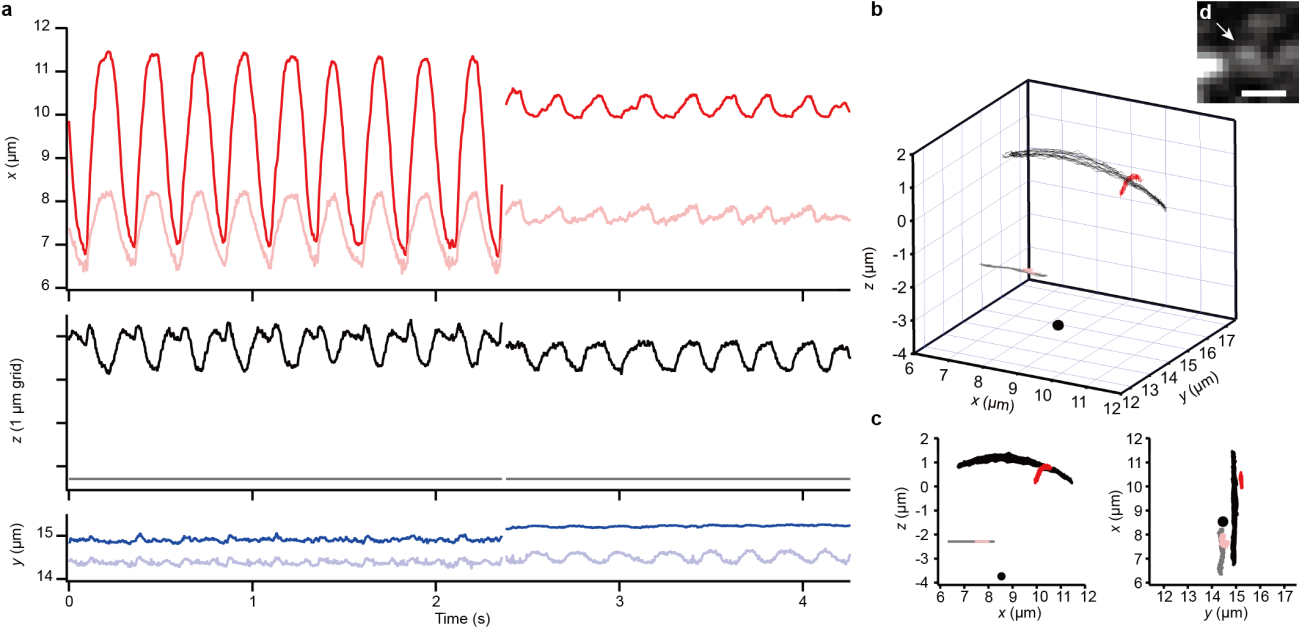


**Supplementary Figure 2** Movement of the tip and middle portion of the cilium. **a** A typical example of the time course of the movement in *x*, *y* and *z* directions at 100 µm ATP. Red, pink, black, gray, blue and sky blue traces represent the trajectories of the tip in *x*, the middle in *x*, the tip in *z*, the middle in *z*, the tip in *y* and the middle in *y* directions respectively. **b** 3-D plots. Black, red, gray, pink lines represent the trajectories of the tip during beating, the tip during trapping, the middle during beating and the middle during trapping respectively. The black sphere represents the theoretical adhesion point of the end of a cilium to the grass surface. **c** Trajectories on the *xz* and *yx* planes. **d** The image of the adhesion point of the end of a cilium to the glass surface obtained using TIRFM (*arrow*). Scale bar, 2 µm.


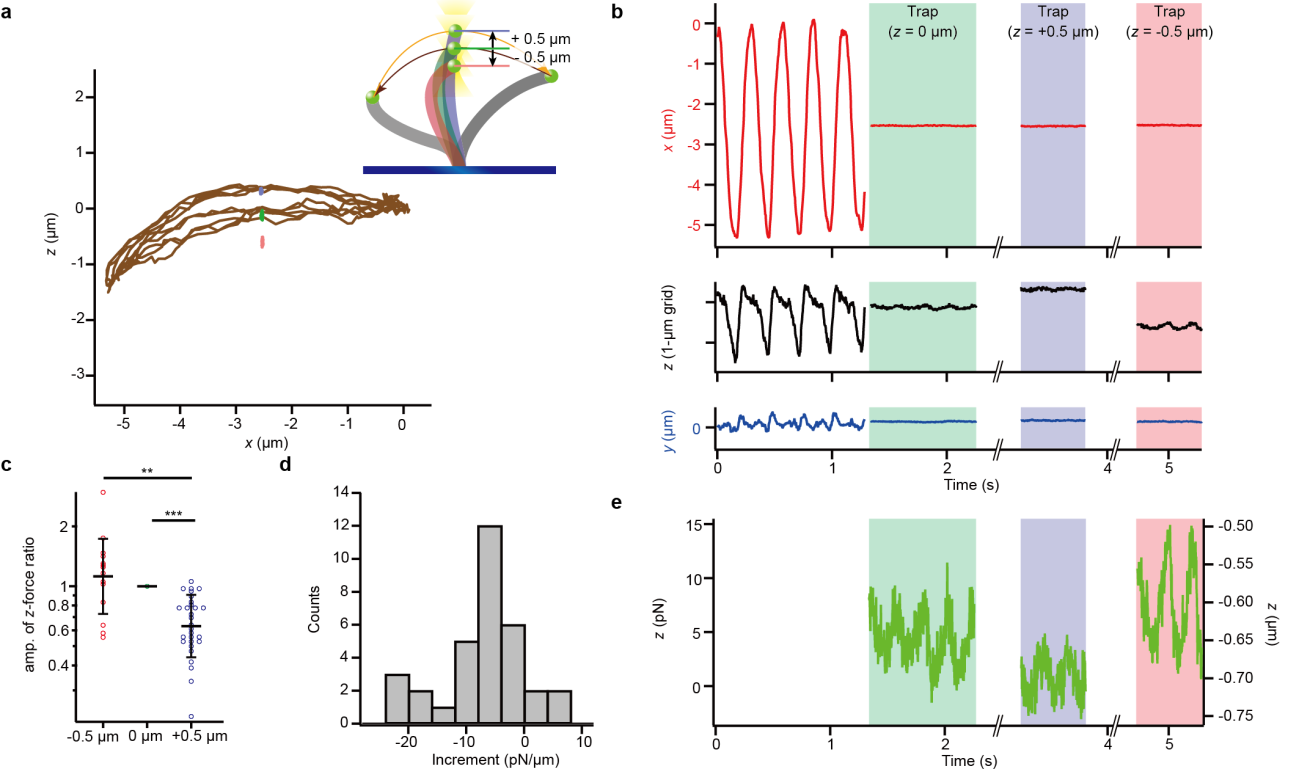


**Supplementary Figure 3** Optical trapping of the tip of the single cilium beating under the external longitudinal force. **a** A typical example of trajectories in the *x-z* plane of an end-attached bead during beating. Brown, the trajectory under no load. Green, the trajectory of the bead under trapping. Blue and red respectively represent trajectories of the bead when the trapping center was displaced with +0.5 µm and -0.5 µm along the *z*-axis. (*inset*) A schematic illustration of the experimental setup. During trapping, the trapping center was displaced with +0.5 µm and -0.5 µm along the *z*-axis. **b** An example of the time course of bead displacement in *x* (*top*), *z* (*middle*) and *y* (*bottom*) directions. **b,e** Green, blue and red areas respectively represent that the original trapping center, displaced with +0.5 µm and displaced with -0.5 µm along the *z*-axis. **c** The ratios of the amplitude of force under the trapping center that is displaced with ±0.5 µm to that under the original trapping center. Medial horizontal lines and error bars represent geometric means and geometric s.d., respectively. ***p*<0.01, ****p*<0.001 two-tailed *t*-test. **d** The increment in the amplitude of *z*-force. The negative value means that the force decreased as the cilium elongated. **e** Magnified view of the time course of the *z*-axial trajectory under trapping conditions.

**File Name: Supplementary Video 1**

**Description:** Recording of a fluorescent image of a bead attached to the tip of the cilium (*left* and *center*) and the DIC image of the middle portion of the cilium (*right*). A bead image was split into two images by the prism located in front of Camera-1, at an equivalent back focal plane of the objective (*left*), and they were superimposed into one image in pseudo-colors of red and blue (*center*). 0 s indicates the onset of laser trapping of the bead.

**File Name: Supplementary Video 2**

**Description:** The DIC image of the inclined cilium. One end of cilium was attached to the coverslip and the other end, the free end, was attached to a 200 nm bead. As observed *in vitro*, the wave propagated from the coverslip adhesion point to the free end.
